# Expression profile of microRNAs in porcine alveolar macrophages after *Toxoplasma gondii* infection

**DOI:** 10.1186/s13071-019-3297-y

**Published:** 2019-01-29

**Authors:** Senyang Li, Jing Yang, Luyao Wang, Fen Du, Junlong Zhao, Rui Fang

**Affiliations:** 1 State Key Laboratory of Agricultural Microbiology, College of Veterinary Medicine, Huazhong Agricultural University, No.1, Shizishan Street, Wuhan, Hubei Province 430070 People’s Republic of China; 2 Hubei Center for Animal Diseases Control and Prevention, Wuhan, Hubei Province 430070 People’s Republic of China

**Keywords:** *Toxoplasma gondii*, microRNA (miRNA), Porcine alveolar macrophages (3D4-21)

## Abstract

**Background:**

*Toxoplasma gondii* is an apicomplexan protozoan parasite that can cause serious clinical illnesses in both humans and animals. microRNAs (miRNAs) are non-protein-coding RNAs that can regulate the expression of target genes. A previous study found that many miRNAs were differentially expressed after *T. gondii* infection and exert significant effects and revealed that both host survival and the virulence of different strains can be regulated by different miRNAs. Macrophages play an important role in *T. gondii* infection, but few studies have investigated the relationship between miRNAs and porcine alveolar macrophages infected with *T. gondii.*

**Methods:**

Porcine alveolar macrophages (3D4-21) were infected with the RH (Type I) and Me49 (Type II) strains of *T. gondii* for 12 h and 24 h and then harvested. miRNA libraries were generated using the NEBNext® Multiplex Small RNA Library Prep Set for Illumina® (NEB, USA), and the miRNA expression levels were estimated based on transcripts per million reads (TPM).

**Results:**

Our study generated six miRNA expression profiles from macrophages infected with RH and Me49 compared with the control groups. The comparison of the *T. gondii*-infected and uninfected samples identified 81 differentially expressed miRNAs, including 36 novel miRNAs and 45 mature miRNAs. The target genes of these differentially expressed miRNAs were predicted using miRanda software, and ssc-miR-127 and ssc-miR-143-3p were predicted to regulate nitric oxide synthase 1 (NOS1) and nitric oxide synthase 3 (NOS3), respectively, which play essential roles in synthesizing nitric oxide (NO) by oxidizing L-arginine. These genes were differentially expressed in both the RH- and Me49-infected groups. A KEGG enrichment analysis indicated that the predicted target genes were involved in multiple signaling pathways, including FcγR-mediated phagocytosis, the AMPK signaling pathway, the mTOR signaling pathway, and the FcγRI signaling pathway, all of which are indispensable for the normal functioning of porcine alveolar macrophages.

**Conclusions:**

Our results provide data on the miRNA profile of porcine alveolar macrophages infected with *T. gondii.* To our knowledge, this study provides the first demonstration of the relationship between miRNA and macrophages of swine origin. Understanding the functions of these regulated miRNAs will aid the investigation of *T. gondii* infectious diseases, and the differentially expressed miRNAs might be candidate drug targets for *T. gondii* infection in pigs.

**Electronic supplementary material:**

The online version of this article (10.1186/s13071-019-3297-y) contains supplementary material, which is available to authorized users.

## Background

*Toxoplasma gondii* is an apicomplexan protozoan parasite that infects all nucleated cells and a widely distributed zoonotic parasite that can cause serious clinical illnesses in animals and humans. Although *T. gondii* has a heteroxenous life-cycle and a broad host range, its sexual reproduction only occurs in the small intestine of felids, the only definitive hosts of *T. gondii*. *Toxoplasma gondii* can cause severe disease in warm-blooded animals, including humans; immunocompetent individuals may experience fever, lymphadenopathy and muscle aches, and immunosuppressed patients may present with neurological symptoms, encephalitis and toxoplasma encephalitis. *Toxoplasma gondii* acquired during pregnancy might cause serious damage to newborn babies, including sight and hearing impairments and central nervous system diseases [1–3]. In many countries, *T. gondii* is one of the major causes of abortion and stillbirth in pig and sheep farms. Pork is the most widely consumed meat per capita in most countries, but pigs pose potential zoonotic risks because they might carry bacteria and other pathogens, including *T. gondii*. In fact, pigs are among the most important sources of material used in human xenoplastic transplantation, and the high rate of *T. gondii* infection in pigs is a huge challenge for future organ transplantations [4, 5]. Therefore, the prevention and control of swine toxoplasmosis is of great significance to animal husbandry and for the prevention of human *T. gondii* infection.

Macrophages are myeloid immune cells that form part of the mononuclear phagocyte system. These cells, which are found in many tissues and organs, including the spleen, lungs and lymph nodes, consume and digest dead cells, debris, and parasites in the body [6, 7]. During inflammation, macrophages are key players in phagocytosis, antigen presentation, and the secretion of various cytokines, chemokines, and growth factors. *Toxoplasma gondii* infection induces macrophages to secrete various cytokines, including the upregulated cytokines IFN-γ, TNF-α, IL-1, IL-2 and IL-12 and the downregulated cytokines IL-4, IL-6 and IL-10 [8]. IFN-γ plays a leading role in the host’s fight against *T. gondii*, and IFN-γ induces an increase in the insecticidal ability of macrophages. During the processes of phagocytosis, macrophage and pathogen surface receptors assimilate into a membrane-bound digestive vacuole; therefore, macrophages are considered an important component of the innate immune system’s first line of defense against pathogen infection [9]. However, many intracellular pathogens exploit this mechanism to ensure their internalization and survival in phagocytes, particularly macrophages [10]. This mechanism is one of the strategies used by *T. gondii* to evade host immunity.

miRNAs are a class of endogenous non-protein-coding RNAs that serve as negative regulators of the host response through the miRNA-induced silencing complex (miRISC) in apicomplexan parasites [11]. Since the discovery of miRNAs in 1993, these molecules have attracted wide attention due to their unique functions and provide a new dimension of molecular biology. Increasing evidence suggests that miRNAs play multiple roles in diverse biological processes, including organ development, cell proliferation and division, pathological processes [12–14] and in *T. gondii* infection. For example, following *T. gondii* infection, the miRNA-30c-1, miRNA-125b-2, miRNA-23b-27b-24-1 and miRNA-17~92 cluster genes bind to the STAT3 promoter in human macrophages, which leads to increased apoptosis of these cells [15]. Early during the *T. gondii* infection process in mice, three miRNAs (mmu-miR-712-3p, mmu-miR-511-5p and mmu-miR-217-5p) are prominently expressed by both the RH and ME49 strains, and the increase in the expression of these miRNAs is specific to *Toxoplasma* species [16].

In our study, porcine alveolar macrophages were infected with the RH and Me49 strains of *T. gondii*. The goal of our study was to explore the changes in miRNA expression in porcine alveolar macrophages after infection with *T. gondii*. A previous study revealed that miRNAs have significant effects at the posttranscriptional level on physiology, pathogenesis, and immunology. Our results provide full miRNA profiles of porcine alveolar macrophages infected with *T. gondii*, and differential miRNA expression was further analyzed by target prediction and pathway analysis. Unveiling the roles of differentially expressed miRNAs in macrophages will help researchers obtain a better understanding of the interactions between *T. gondii* and macrophages. In addition, an understanding of the functions of these regulated miRNAs will aid the investigation of toxoplasmosis, and the differentially expressed miRNAs might be candidate drug targets for *T. gondii* infection in pigs.

## Methods

### Parasite infection and sample collection

The RH (type I) and Me49 (type II) strains of *T. gondii* were cryopreserved in liquid nitrogen in our laboratory. Porcine alveolar macrophages and human foreskin fibroblasts (HFF) were cultured in DMEM with 10% FBS. Porcine alveolar macrophages were infected with the RH and Me49 strains at a macrophage-to-tachyzoite ratio of 1:5 (MOI = 5) to ensure an appropriate infective dose. RH- and Me49-infected macrophages were harvested at 12 and 24 h after infection with *T. gondii* (herein referred to as RH12 and RH24, and M12 and M24), and samples from the control group were collected at the same time points (referred to as D12 and D24). Thus, six samples were generated during the study: RH12, RH24, M12, M24, D12 and D24. RNA was extracted from all samples using TRIzol, and small RNA sequencing was then performed to characterize the global miRNA transcriptional response to *T. gondii* infection.

### RNA quantification and qualification

The degradation and contamination of RNA were monitored using 1% agarose gels. The RNA purity was checked using a NanoPhotometer® spectrophotometer (Implen, GER), and the RNA concentration was measured using a Qubit® RNA Assay Kit with a Qubit® 2.0 Fluorometer (Life Technologies, USA). The RNA integrity was assessed using an RNA Nano 6000 Assay Kit with an Agilent Bioanalyzer 2100 system (Agilent Technologies, USA).

### Preparation of libraries for small RNA sequencing and data analysis

The samples were sent to the Beijing Novogene Bioinformatics Institute for Illumina sequencing. Three micrograms of total RNA was used as the input material to generate a small RNA library used for the analysis of miRNAs. Sequencing libraries were generated using the NEBNext® Multiplex Small RNA Library Prep Set for Illumina® (NEB), and first-strand cDNA was then synthesized using Moloney Murine Leukemia Virus Reverse Transcriptase (M-MuLV-RT). PCR amplification was performed using LongAmp Taq 2× Master Mix, and the PCR products were purified on an 8% polyacrylamide gel. The libraries obtained from the different samples were sequenced on an Illumina HiSeq 2500/2000 platform, and 50-bp single-end reads were generated.

The raw data (raw reads) in FASTQ format were first processed using Python and custom Perl scripts. Clean data (clean reads) were obtained after removing various reads, including poly-N reads, reads without a 3' adapter or an insert tag, reads with 5' adapter contamination, reads with poly A, T, G or C and low-quality reads from the raw data. Moreover, the Q20, Q30 and GC contents of the raw data were calculated. We then selected a specific length for the clean reads to perform all subsequent analyses.

To analyze the expression and distribution of the small RNA tags on the reference sequence, we mapped the small RNA tags to the reference sequence using Bowtie [17] without allowing any mismatch; this process was also used to search for known miRNAs. miRBase20.0 was used as a reference, and potential miRNA and secondary structures were obtained using modified mirdeep2 [18] and srna-tools-cli software. Custom scripts were used to obtain the miRNA counts as well as the base bias on the first positions of the identified miRNAs with a specific length and on each position of all identified miRNAs. All small RNA tags were mapped to the Rfam database, Repeat Masker or the various types of data from the specified species to remove tags originating from repeat sequences, protein-coding genes, rRNAs, snRNAs, tRNAs and snoRNAs.

Novel miRNAs can be predicted based on the hairpin structure of the miRNA precursor. To predict novel miRNAs, we explored the Dicer cleavage site, the secondary structure and the minimum free energy of the small RNA tags that were not annotated in the former steps using the available software mirdeep2 [18] and miREvo [19].

The target genes of the miRNAs were predicted using miRanda software. The measure transcripts per million (TPM) was used to estimate miRNA expression levels based on the following criteria: normalized expression = mapped read count/total reads*1,000,000 [20]. Differential expression analysis between two groups was performed using the *DESeq* R package (v.1.8.3). *P*-values were adjusted using the Benjamini & Hochberg method, and by default, a corrected *P*-value of 0.05 was set as the threshold for significant differential expression. The target gene candidates of differentially expressed miRNAs were subjected to gene ontology (GO) enrichment analysis. The statistical enrichment of the target gene candidates in KEGG pathways was tested using KEGG Orthology Based Annotation System (KOBAS) software.

### Validation of miRNA expression

The results of the miRNA expression analysis were validated by tailing-reaction quantitative RT-PCR using an ABI StepOne Sequence Detection System with SYBR Green qPCR Super Mix according to the manufacturer’s recommended protocol (Invitrogen, USA). All qRT-PCR reactions were performed in triplicate, and snRNA U6 was used as an internal control for the normalization and quantification of miRNA expression.

## Results

### RNA quality testing

All RNAs were subjected to quality testing to ensure their availability. The detected contents included OD260/280, OD260/230, 28S/18S and RNA integrity (RIN), and all test results were qualified.

### miRNA expression profiles of macrophages after *T. gondii* infection

Our study generated six miRNA expression profiles from macrophages infected with either RH or Me49 or non-infected macrophages (control) at two different time points, and more than ten million raw sequence reads from each group were mapped to the sRNA library. In addition, more than ten million high-quality clean reads were obtained after low-quality reads, junk sequences and adaptor sequences were removed from the raw sequence reads. The sequencing data are summarized in Tables 1 and 2.

**Table 1:** Summary of small RNA sequencing data

Library type	Reads	Bases	Error rate (%)	Q20 (%)	Q30 (%)	GC content (%)
Me49-12	13,132,137	0.657G	0.02	91.99	85.52	51.23
Control-12	13,136,212	0.657G	0.02	92.50	86.08	48.16
RH-12	10,468,948	0.523G	0.01	96.93	93.45	51.59
Me49-24	13,959,111	0.698G	0.02	94.22	89.18	52.52
Control-24	14,751,563	0.738G	0.02	94.47	89.72	47.80
RH-24	14,931,863	0.747G	0.02	93.28	88.06	52.41

**Table 2:** Summary of the standard bioinformatics quality checks and cleaning of small RNAs

Library type	Me49-12	Control-12	RH-12	Me49-24	Control-24	RH-24
Total reads	13,132,137	13,136,212	10,468,948	13,959,111	14,751,563	14,931,863
N% > 10%	1	2	57	69	75	28
Reads with 5-adapter-contamination	2381	716	2202	2433	544	2617
Reads with ploy A/T/G/C	44,095	21,489	41,015	29,190	11,655	60,839
Low-quality reads	82,182	78,823	22,801	2,797,182	2,610,836	3,360,947
3-adapter- null or insert-null reads	325,730	238,506	143,336	235,442	161,513	228,911
Clean reads (%)	12,677,748 (96.54)	12,796,676 (97.42)	10,259,537 (98.00)	10,894,795 (78.05)	11,966,940 (81.12)	11,278,521 (75.53)

**Fig. 1:**
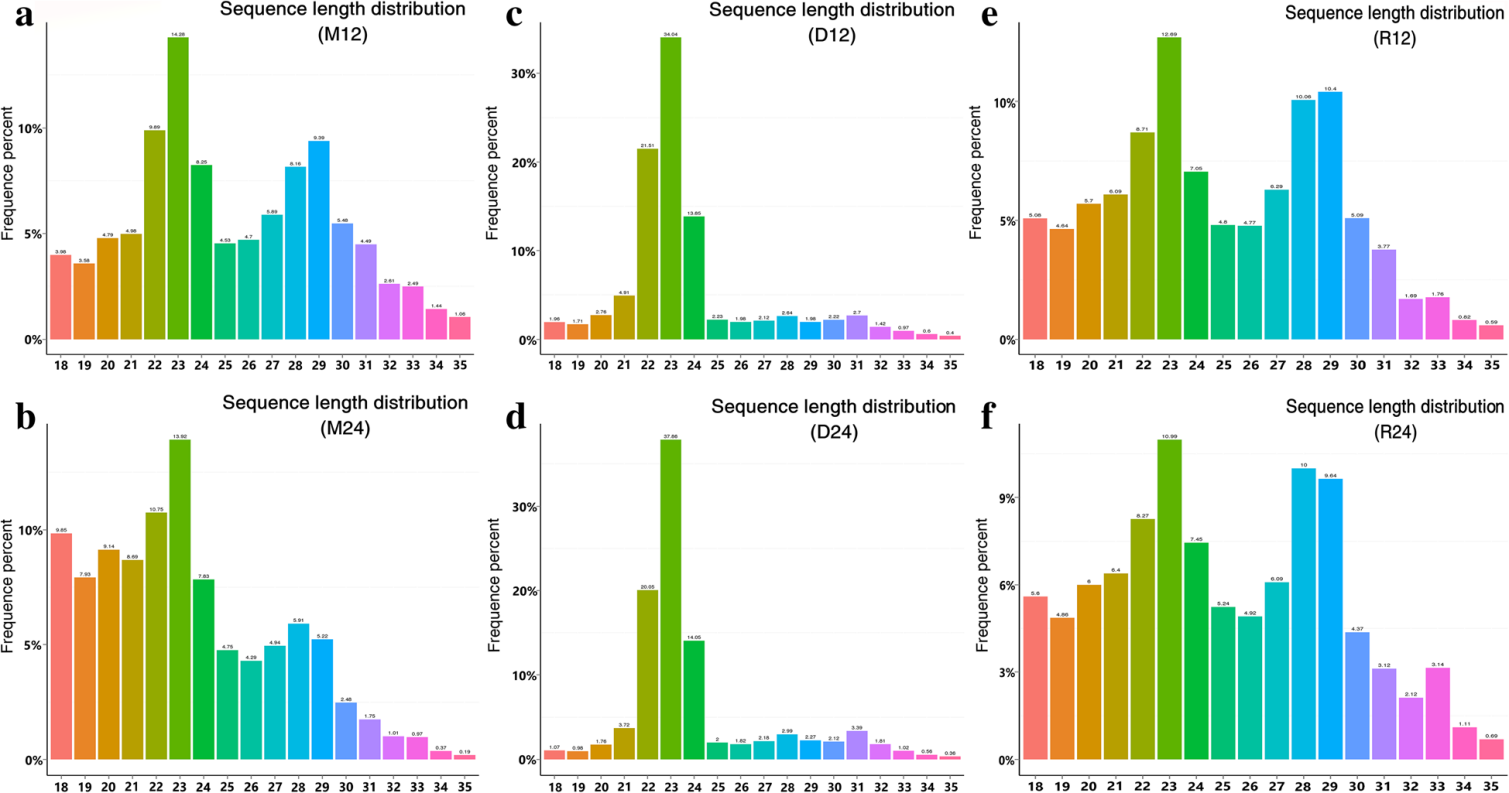
Length distribution of the small RNAs expressed in porcine alveolar macrophages infected or not infected with *T. gondii*. **a**, **b** Macrophages harvested 12 h (M12) and 24 h (M24) after infection with the Me49 strain. **c**, **d** Uninfected macrophages (D12 and D24, control groups).**e**, **f** Macrophages harvested 12 h (R12) and 24 h (R24) after infection with the RH strain

Most (> 70%) of the total clean reads in the control group had a length of 22–24 nucleotides, and the length of the clean reads was significantly changed after *T. gondii* infection. Compared with the control group, the number of sRNAs between 22–24 nucleotides in length decreasing by 50% in experimental groups, and the length of the majority of the sRNAs in the experimental groups was 19–29 nucleotides (Fig. 1a-d). We removed sRNAs of 24–29 nucleotides and 32–35 nucleotides, and the remaining clean reads were used for the identification of mature miRNAs and for the prediction of novel miRNAs.

The comparison of the *T. gondii*-infected and uninfected group revealed 81 differentially expressed miRNAs, including 36 novel miRNAs and 45 mature miRNAs. In the group infected with the RH strain, 19 mature miRNAs were upregulated and nine mature miRNAs were downregulated. In addition, 33 novel miRNAs were identified in this group, all of which were upregulated (Tables 3, 4). Moreover, an analysis of the Me49-infected group revealed that 13 and six mature miRNAs were upregulated and downregulated, respectively, and that all 23 novel miRNAs detected were upregulated (Tables 5, 6). Detailed miRNA data for the different groups (M12, M24, R12 and R24) are summarized in Additional file 1: Table S1.

**Table 3:** Differential expression of mature miRNAs in porcine alveolar macrophages infected with the RH strain

Type	miRNA	Fold change	*P*-value	*q*-value
Upregulated	ssc-miR-451	1.949	3.51E-75	4.53E-74
	ssc-miR-1285	4.9342	7.74E-72	8.04E-71
	ssc-miR-4332	10.907	6.13E-59	5.45E-58
	ssc-miR-146a-5p	1.1608	7.46E-32	4.43E-31
	ssc-miR-9	4.0498	1.27E-28	7.66E-28
	ssc-miR-9-1	4.0498	1.27E-28	7.66E-28
	ssc-miR-4332	8.209	6.69E-22	3.66E-21
	ssc-miR-99a	3.6665	7.54E-21	3.89E-20
	ssc-miR-542-3p	1.2399	2.26E-20	1.13E-19
	ssc-miR-1285	3.1429	7.26E-17	3.37E-16
	ssc-miR-199a-3p	4.4496	1.59E-16	7.00E-16
	ssc-miR-199b-3p	4.4496	1.59E-16	7.00E-16
	ssc-miR-4331	3.8591	8.24E-08	1.90E-07
	ssc-miR-486	1.4972	6.85E-07	1.87E-06
Downregulated	ssc-miR-125b	-1.0558	1.62E-97	2.44E-96
	ssc-miR-1	-1.0748	7.10E-23	3.69E-22
	ssc-miR-335	-1.1053	1.45E-20	6.69E-20
	ssc-let-7c	-1.0411	1.39E-11	5.47E-11
	ssc-miR-99a	-4.0677	3.94E-09	9.82E-09
	ssc-miR-331-3p	-1.0933	0.0011139	0.0022359
	ssc-miR-421-3p	-1.6714	0.002109	0.0029857
	ssc-miR-17-3p	-4.4202	0.0023737	0.0032496
	ssc-miR-362	-1.0508	0.0057743	0.0067864

**Table 4:** Differential expression of novel miRNAs in macrophages infected with the RH strain

Type	miRNA	Fold change	*P*-value	*q*-value
Upregulated	novel_417	5.6453	0	0
	novel_72	8.4105	1.19E-262	5.37E-261
	novel_90	6.8889	7.10E-109	1.17E-107
	novel_394	5.0994	5.07E-16	1.91E-15
	novel_382	6.1533	4.66E-11	1.32E-10
	novel_132	5.7071	8.68E-10	2.25E-09
	novel_486	5.6678	2.15E-08	5.15E-08
	novel_334	5.5158	1.06E-07	2.40E-07
	novel_511	5.3459	5.36E-07	1.13E-06
	novel_594	5.3459	5.36E-07	1.13E-06
	novel_601	5.3459	5.36E-07	1.13E-06
	novel_558	5.1533	2.81E-06	5.46E-06
	novel_601	5.303	7.32E-06	1.86E-05
	novel_454	1.3852	9.05E-06	1.71E-05
	novel_578	4.1429	1.03E-05	2.59E-05
	novel_591	4.9309	1.52E-05	2.79E-05
	novel_591	5.062	4.14E-05	9.58E-05
	novel_380	4.6678	8.59E-05	0.0001466
	novel_473	4.6678	8.59E-05	0.0001466
	novel_394	3.406	0.0001544	0.0003401
	novel_290	3.4441	0.0002395	0.0003776
	novel_578	3.4441	0.0002395	0.0003776
	novel_471	1.4744	0.0004257	0.0006314
	novel_511	4.41	0.0014925	0.0029629
	novel_384	5.2921	0.0021461	0.003004
	novel_515	1.5602	0.0023622	0.0032496
	novel_305	3.9309	0.0031796	0.0040836
	novel_449	3.9309	0.0031796	0.0040836
	novel_451	3.9309	0.0031796	0.0040836
	novel_541	3.9309	0.0031796	0.0040836
	novel_550	3.9309	0.0031796	0.0040836
	novel_564	3.9309	0.0031796	0.0040836
	novel_382	4.1876	0.0037708	0.0069513
Downregulated	none	none	none	none

**Table 5:** Differential expression of mature miRNAs in macrophages infected with the Me49 strain

Type	miRNA	Fold change	*P*-value	*q*-value
Upregulated	ssc-miR-451	2.1667	6.36E-118	6.93E-116
	ssc-miR-1285	4.4641	5.84E-46	5.09E-45
	ssc-miR-122	1.6617	1.14E-19	1.77E-18
	ssc-miR-486	1.9641	3.28E-15	3.70E-14
	ssc-miR-4332	6.8595	4.57E-11	4.40E-10
	ssc-miR-99a	1.1177	3.86E-07	8.60E-07
	ssc-miR-143-3p	1.3819	5.90E-06	1.16E-05
	ssc-miR-126-3p	1.2962	9.48E-06	1.75E-05
	ssc-miR-17-3p	1.8355	9.56E-06	1.75E-05
	ssc-miR-199a-3p	1.784	2.25E-05	3.80E-05
	ssc-miR-199b-3p	1.784	2.25E-05	3.80E-05
	ssc-miR-4331	3.3689	4.53E-05	6.96E-05
	ssc-miR-127	2.4514	0.00037218	0.00049907
Downregulated	ssc-miR-222	-1.0298	4.35E-161	1.14E-159
	ssc-miR-1	-1.2042	4.12E-38	3.08E-37
	ssc-miR-1296-5p	-4.7387	0.00036318	0.00049334
	ssc-miR-935	-2.4384	0.0011422	0.0014097
	ssc-miR-421-3p	-1.5242	0.0022573	0.0026234

**Table 6:** Differential expression of novel miRNAs in macrophages infected with the Me49 strain

Type	miRNA	Fold change	*P*-value	*q*-value
Upregulated	novel_417	3.3666	1.33E-117	1.09E-115
	novel_72	6.8454	1.78E-109	1.17E-107
	novel_90	5.758	2.69E-56	8.00E-55
	novel_558	6.1076	2.49E-10	7.65E-10
	novel_132	5.784	2.13E-09	6.03E-09
	novel_394	4.3689	4.98E-09	1.34E-08
	novel_451	5.8446	6.85E-09	1.75E-08
	novel_374	5.6381	6.64E-08	1.54E-07
	novel_601	5.6381	6.64E-08	1.54E-07
	novel_591	5.2596	2.24E-06	4.58E-06
	novel_511	5.1076	7.46E-06	1.44E-05
	novel_578	3.9539	1.82E-05	3.16E-05
	novel_382	4.9377	2.54E-05	4.02E-05
	novel_541	4.9377	2.54E-05	4.02E-05
	novel_594	4.9377	2.54E-05	4.02E-05
	novel_515	1.9997	0.00022197	0.00030955
	novel_409	3.5913	0.00023715	0.00032638
	novel_284	4.2596	0.0011456	0.0014097
	novel_334	4.2596	0.0011456	0.0014097
	novel_380	4.2596	0.0011456	0.0014097
	novel_421	2.1763	0.0031476	0.0035023
	novel_486	3.9377	0.0043094	0.0046468
	novel_547	3.9377	0.0043094	0.0046468
Downregulated	none	none	none	none

**Fig. 2:**
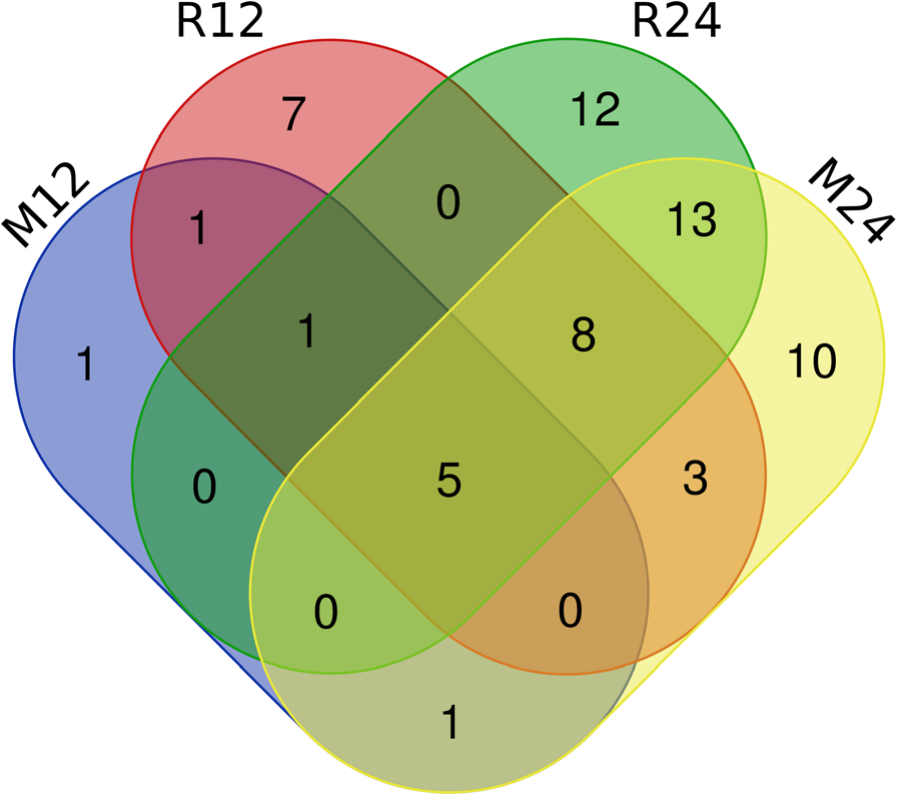
Venn diagram showing the number of overlapping differentially expressed miRNAs in the different groups

An analysis of the miRNAs in the different groups is shown in Fig. 2. The comparison of the porcine alveolar macrophages infected with RH and Me49 for 12 h with the corresponding control group identified seven miRNAs (ssc-miR-486, ssc-miR-451, ssc-miR-4332, ssc-miR-1285, ssc-novel-90, ssc-novel-72 and ssc-novel-417) that were differentially expressed in both infected groups. Moreover, an analysis of groups obtained 24 h after infection revealed 26 miRNAs that were differentially expressed in the RH- and Me49-infected groups compared with the control. In total, five miRNAs (ssc-miR-4332, ssc-miR-1285, ssc-novel-90, ssc-novel-72 and ssc-novel-417) were differentially expressed in the M12, M24, R12 and R24 groups compared with the control group, and these detailed miRNA data are summarized in Table 7.

**Table 7:** Summary of the overlapping differentially expressed miRNAs in the different groups

Different group	Quantity	Common differentially expressed miRNAs
M12, M24, R12, R24	5	ssc-miR-4332, ssc-miR-1285, ssc-novel-90 ssc-novel-72, ssc-novel-417
M12, M24	6	ssc-miR-199a-5p, ssc-miR-4332, ssc-miR-1285, ssc-novel-417, ssc-novel-90, ssc-novel-72
R12, R24	14	ssc-miR-451, ssc-miR-99a, ssc-miR-127, ssc-miR-1285, ssc-miR-4332, ssc-novel-72, ssc-novel-90, ssc-novel-591, ssc-novel-382, ssc-novel-601, ssc-novel-511, ssc-novel-394, ssc-novel-578, ssc-novel-417
M12 R12	7	ssc-miR-1285, ssc-miR-486, ssc-miR-451, ssc-novel-417, ssc-miR-4332, ssc-novel-72, ssc-novel-90
M24, R24	25	ssc-miR-1, ssc-miR-127, ssc-miR-4331, ssc-miR-421-3p, ssc-miR-17-3p, ssc-miR-4332, ssc-miR-1285, ssc-miR-99a, ssc-novel-515, ssc-novel-132, ssc-novel-601, ssc-novel-594, ssc-novel-380, ssc-novel-334, ssc-novel-394, ssc-novel-451, ssc-novel-382, ssc-novel-511, ssc-novel-90, ssc-novel-558, ssc-novel-72, ssc-novel-591, ssc-novel_486, ssc-novel-578, ssc-novel-541, ssc-novel-417

### GO and KEGG analyses

**Fig. 3:**
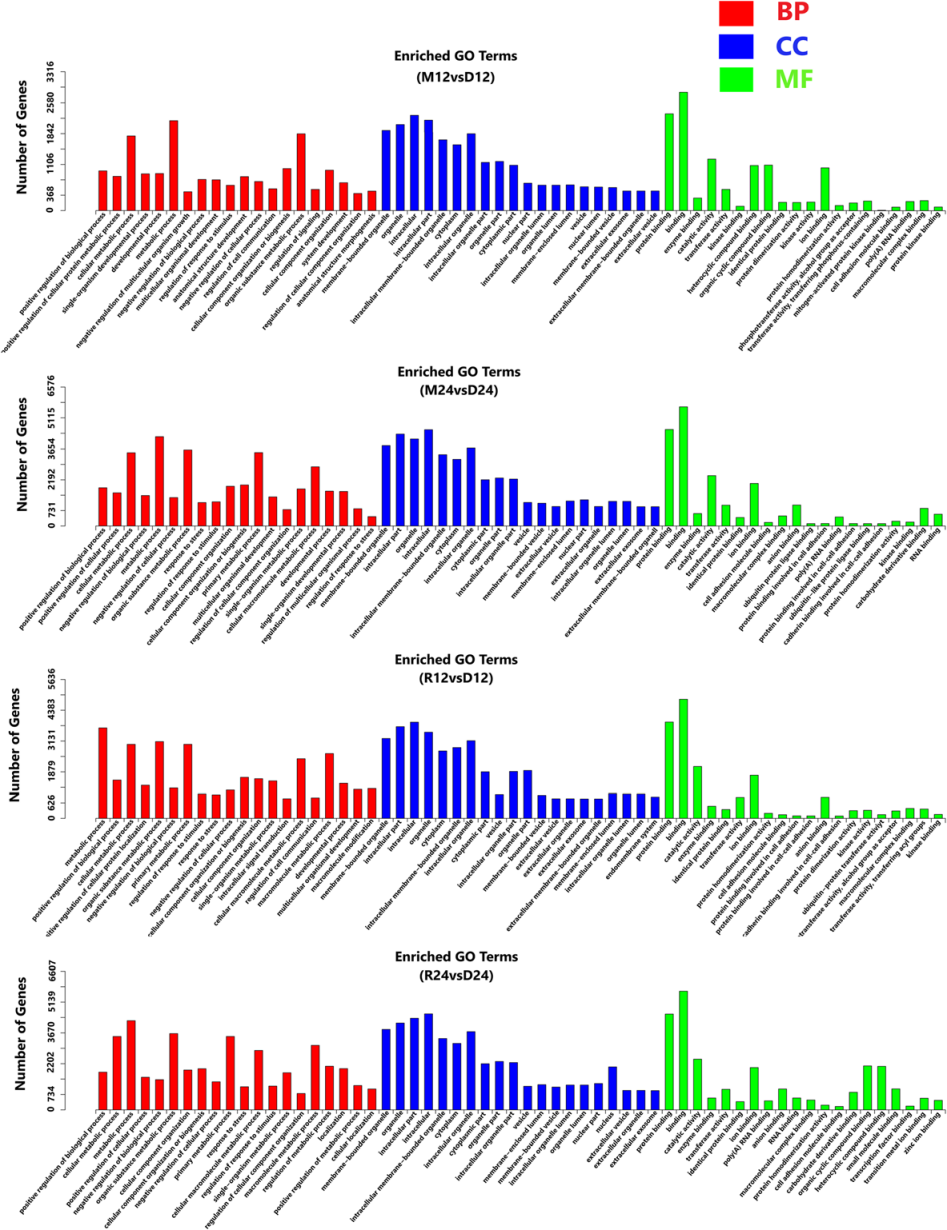
GO analysis of macrophages infected or uninfected with *T. gondii*. **a**, **b** Macrophages harvested 12 h or 24 h after infection with the Me49 strain. **c**, **d** Macrophages harvested 12 h or 24 h after infection with the RH strain. *Abbreviations*: BP, biological process; CC, cellular component; MF, molecular function

In total, 7462 predicted targets were identified (Additional file 2: Table S2). A gene ontology (GO) enrichment analysis was the performed based on the predicted target genes of the differentially expressed miRNAs to identify the enriched biological processes, molecular functions and cellular components. The enriched GO terms of the cellular component, molecular function and biological process categories are shown in Fig. 3. A KEGG enrichment analysis demonstrated that the target genes were involved in multiple signaling pathways, including FcγR-mediated phagocytosis, AMPK signaling, mTOR signaling, phosphatidylinositol signaling, Fcγ epsilon RI signaling, B cell receptor signaling, T cell receptor signaling, pathways in cancer and VEGF signaling. The top 20 KEGG enrichment pathways are shown in Tables 8 and 9.

**Table 8:** Top 20 enriched pathways after infection with the RH strain for 12 h (R12) and 24 h (R24)

R12 *vs* D12	R24 *vs* D24
Pancreatic cancer	Chagas disease (American trypanosomiasis)
T cell receptor signaling pathway	Pancreatic cancer
Chagas disease (American trypanosomiasis)	Acute myeloid leukemia
mTOR signaling pathway	Malaria
Acute myeloid leukemia	Chronic myeloid leukemia
Phosphatidylinositol signaling system	Proteoglycans in cancer
Platelet activation	Endometrial cancer
Inositol phosphate metabolism	Rheumatoid arthritis
Proteoglycans in cancer	Fc gamma R-mediated phagocytosis
Chronic myeloid leukemia	Inositol phosphate metabolism
Inflammatory bowel disease (IBD)	VEGF signaling pathway
B cell receptor signaling pathway	TNF signaling pathway
Endometrial cancer	Phosphatidylinositol signaling system
Amoebiasis	mTOR signaling pathway
VEGF signaling pathway	Toll-like receptor signaling pathway
Renal cell carcinoma	Fc epsilon RI signaling pathway
Insulin signaling pathway	Thyroid hormone signaling pathway
TNF signaling pathway	Butanoate metabolism
Fc epsilon RI signaling pathway	Non-small cell lung cancer

**Table 9:** Top 20 enriched pathways after infection with the Me49 strain for 12 h (M12) and 24 h (M24)

M12 *vs* D12	M24 *vs* D24
Pancreatic cancer	Chagas disease (American trypanosomiasis)
Chronic myeloid leukemia	Pancreatic cancer
Fc gamma R-mediated phagocytosis	Thyroid hormone signaling pathway
Pathways in cancer	Renal cell carcinoma
Acute myeloid leukemia	T cell receptor signaling pathway
Renal cell carcinoma	TNF signaling pathway
Insulin signaling pathway	mTOR signaling pathway
Proteoglycans in cancer	Fc gamma R-mediated phagocytosis
Chagas disease (American trypanosomiasis)	Proteoglycans in cancer
Cell cycle	Acute myeloid leukemia
Phosphatidylinositol signaling system	Endometrial cancer
mTOR signaling pathway	Fc epsilon RI signaling pathway
Type II diabetes mellitus	HIF-1 signaling pathway
HTLV-I infection	FoxO signaling pathway
Adherens junction	Chronic myeloid leukemia
Colorectal cancer	Inositol phosphate metabolism
AMPK signaling pathway	Apoptosis
Small cell lung cancer	Phosphatidylinositol signaling system
Axon guidance	Epstein-Barr virus infection

### Verification of differentially expressed miRNAs by real-time fluorescent quantitative PCR

**Fig. 4:**
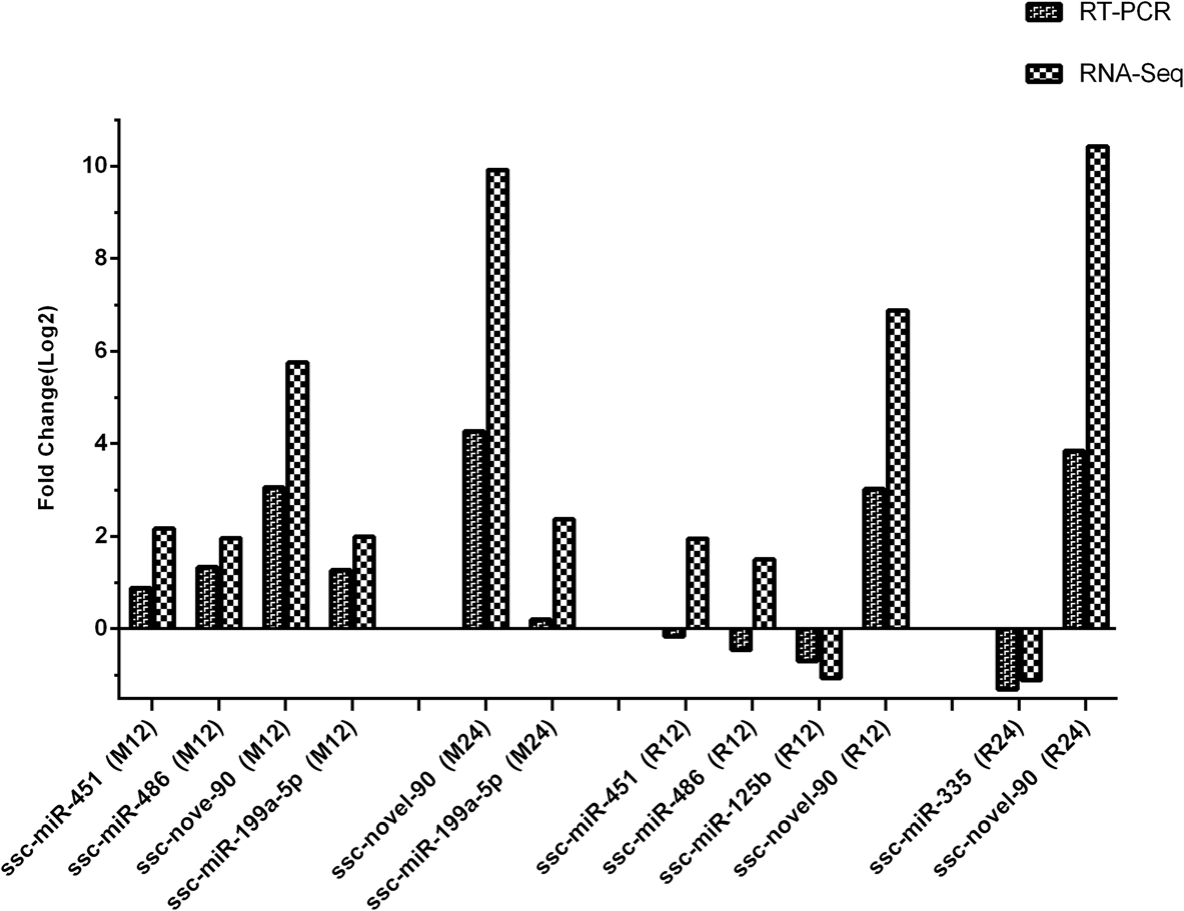
Validation of the selected mature and novel miRNAs by quantitative real-time PCR. M12, M24, R12 and R24 represent the different group

Five mature miRNAs (ssc-miR-335, ssc-miR-199-5p, ssc-miR-125b, ssc-miR-451 and ssc-miR-486) and one novel miRNA (ssc-novel-90) were randomly selected from the set of identified differentially expressed miRNAs, and their expression levels were verified by qRT-PCR using miRNA-specific primers. The expression trends obtained for all the miRNAs by qRT-PCR were similar to those found with the original small RNA-sequencing data (Fig. 4). The results indicated a low false-discovery rate with respect to the RNA-sequencing data and supported the viability of the miRNA expression profiling study. The RNA-sequencing data obtained in our study have been uploaded to the GEO database with the accession number GSE119643.

## Discussion

Previous studies have shown that miRNAs play a critical role in host cells, and many miRNAs are up- or downregulated after infection with *T. gondii* [15, 16]. We used small RNA sequencing and qRT-PCR approaches to analyze the miRNA expression profiles of porcine alveolar macrophages after *T. gondii* infection. Our results showed that miRNAs are differentially expressed between *T. gondii*-infected and *T. gondii*-uninfected porcine alveolar macrophages, regardless of whether the RH or Me49 strain is used for the infection. We compared the miRNA profiles of *T. gondii*-infected and *T. gondii*-uninfected mice to confirm that the differentially expressed miRNAs might be important in macrophage resistance to *T. gondii* infection. Compared with the control group, 19 and nine mature miRNAs were upregulated and downregulated, respectively, in the RH-infected group, whereas in the Me49-infected group, 13 and six mature miRNAs were upregulated and downregulated, respectively. The fact that all the differentially expressed novel miRNAs were upregulated and most novel miRNAs were only predicted in the experimental group indicate that the miRNAs expression profile was changed significantly after *T. gondii* infection.

The potential target genes of the identified miRNAs were predicted using miRanda software, and this analysis identified 7462 predicted target genes. Previous research has shown that macrophages play a crucial role in the course of *T. gondii* infection because nitric oxide (NO) is produced to control the multiplication of *T. gondii* in activated macrophages both *in vitro* and *in vivo* [21, 22]. Among the differentially expressed miRNAs, ssc-miR-127 and ssc-miR-143-3p were predicted to regulate nitric oxide synthase 1 (NOS1) and nitric oxide synthase 3 (NOS3), respectively, and these predicted targets play essential roles in NO synthesis by oxidizing L-arginine [23] and were differentially expressed in both the RH- and Me49-infected groups. Based on these findings, we can reasonably conclude that ssc-miR-127 and ssc-miR-143-3p might be involved in macrophage resistance to *T. gondii*.

The potential functions and relationships of the identified target genes were predicted through a KEGG pathway enrichment analysis. An analysis of the top 30 KEGG pathways that were found to be enriched in the infected groups revealed many commonalities: the target genes of the differentially expressed miRNAs were mostly enriched in FcγR-mediated phagocytosis, the mTOR signaling pathway and the TNF signaling pathway.

FcγR-mediated phagocytosis plays an important role in defense against parasites through the processes of antigen recognition and phagocytosis in macrophages. SYK kinases are essential in the process of FcγR-mediated phagocytosis and are considered crucial for downstream biochemical processes, including PIP2 breakdown and PLCγ and PI3-kinase activation [24, 25]. The activation of the FcγR signaling pathway requires the PI3-kinase isoform PI3Kβ, members of the Rac small GTPase families and Vav guanine nucleotide exchange factors, and Rac2 and Vav3 in particular play notable roles [26–28]. Five aberrantly expressed miRNAs were found to be related to these critical genes: ssc-miR-127 was predicted to regulate SYK; ssc-miR-421-3p was predicted to regulate Rac2; and ssc-miR-143-3p, ssc-miR-199a-5p and ssc-miR-1285 were predicted to regulate Vav3. Therefore, we can reasonably conclude that by binding to their target genes, the abnormally expressed miRNAs participate in the elimination of *T. gondii* by macrophages.

The TNF signaling pathway has a wide range of functions in innate and adaptive immunity. The signal transduction network of TNF comprises many crosslinked pathways, including the MAPK signaling pathway, the NF-κB signaling pathway and the PI3K signaling pathway. TNF, TNF receptor and downstream members form an immense signaling pathway network. Through binding to TNFR1, TNF receptor activation factor-2 (TRAF2) activates the classical NF-κB pathway to produce cytokines such as IFN-γ, TNF-α, IL-1, IL-2 and IL-12, and these cytokines play a vital role in macrophage resistance to *T. gondii* infection [29–31]. Moreover, the activation of TNFR2 by TNF leads to the recruitment of TRAF2 and the downstream activation of the JNK or p38/MAPK pathways, and the activation of these signal transduction pathways enhances the production of intracellular cytokines. Four aberrantly expressed miRNAs were predicted to be related to the members of these signal transduction pathways: ssc-novel_72 was predicted to regulate TNFR1, and ssc-miR-125b and ssc-miR-143-3p were predicted to regulate TRAF2/5. Thus, these miRNAs might play a crucial role in TNF signaling.

There are a few reports on macrophages and miRNAs in *T. gondii* infection. According to these reports, in human macrophages infected with the atypical genotype China 1 (ToxoDB#9), miR-30c-1, miR-125b, miR-23b-27b-24-1 and the miR-17~92 cluster bind to the STAT3 promoter, which increases the apoptosis of host cells [5, 16]. However, the comparison of the miRNA expression profiles obtained in the present study with the above-mentioned list of miRNAs revealed miR-125b as the only differentially expressed miRNA found in both groups. Moreover, in our study, miR-125b was found downregulated after infection with the RH strain, which indicates that macrophages infected with different genotypes of *T. gondii* present different miRNA expression profiles.

## Conclusions

In conclusion, our study provides significant experimental data on the expression profile of miRNAs in porcine alveolar macrophages infected with *T. gondii.* To our knowledge, this study also provides the first detection of the relationship between miRNA and macrophages of swine origin. Previous research showed that macrophages play a key role in resistance to *T. gondii* infection and that miRNAs play an important role in parasitic infections. Unveiling the roles of differentially expressed miRNAs in macrophages will provide a strong foundation for a more in-depth understanding of the interactions between *T. gondii* and macrophages. Understanding the functions of these regulated miRNAs will aid future investigations of *T. gondii* infectious diseases, and the differentially expressed miRNAs might be candidate drug targets for *T. gondii* infection in pigs.

## Additional files

Additional file 1: **Table S1.** Detailed miRNA data for the different groups (M12, M24, R12, and R24). (XLSX 16 kb)

Additional file 2: **Table S2.** List of miRNA targets identified by miRanda. (XLSX 1247 kb)

## Abbreviations

DMEM: Dulbecco’s modified Eagle’s medium
FBS: Fetal bovine serum
GO: Gene ontology
IFN-γ: Interferon-γ
IL-1: Interleukin-1
IL-10: Interleukin-10
IL-12: Interleukin-12
IL-2: Interleukin-2
IL-4: Interleukin-4
IL-6: Interleukin-6
KEGG: Kyoto Encyclopedia of Genes and Genomes
MOI: Multiplicity of infection
NO: Nitric oxide
NOS1: Nitric oxide synthase 1
NOS3: Nitric oxide synthase 3
qRT-PCR: Quantitative reverse transcription real-time polymerase chain reaction
STAT3: Signal transducer and activator of transcription 3
TNFR2: Tumor necrosis factor receptor 2
TNF-α: Tumor necrosis factor
TPM: Transcripts per million reads
TRAF2/5: TNF receptor-associated factor 2/5
